# Gastroduodenal Ulcers and ABO Blood Group: the Japan Nurses’ Health Study (JNHS)

**DOI:** 10.2188/jea.JE20160204

**Published:** 2018-01-05

**Authors:** Lobna Alkebsi, Yuki Ideno, Jung-Su Lee, Shosuke Suzuki, Junko Nakajima-Shimada, Hiroshi Ohnishi, Yasunori Sato, Kunihiko Hayashi

**Affiliations:** 1 Department of Laboratory Sciences, Graduate School of Health Sciences, Gunma University, Gunma, Japan; 2 Big Data Centre for Integrative Analysis, Gunma University Initiative for Advanced Research, Gunma, Japan; 3 Department of Health Promotion Science, Graduate School of Medicine, The University of Tokyo, Tokyo, Japan; 4 Professor Emeritus, Gunma University and NPO International Eco-Health Research Group, Gunma, Japan; 5 Department of Molecular and Cellular Parasitology, Graduate School of Health Sciences, Gunma University, Gunma, Japan; 6 Department of Global Clinical Research, Graduate School of Medicine, Chiba University, Chiba, Japan; 7 Department of Basic Medical Sciences, Graduate School of Health Sciences, Gunma University, Gunma, Japan

**Keywords:** ABO blood group, gastroduodenal ulcer, Japan Nurses’ Health Study

## Abstract

**Background:**

Although several studies have shown that blood type O is associated with increased risk of peptic ulcer, few studies have investigated these associations in Japan. We sought to investigate the association between the ABO blood group and risk of gastroduodenal ulcers (GDU) using combined analysis of both retrospective and prospective data from a large cohort study of Japanese women, the Japan Nurses’ Health Study (JNHS; *n* = 15,019).

**Methods:**

The impact of the ABO blood group on GDU risk was examined using Cox regression analysis to estimate hazard ratios (HRs) and 95% confidence intervals (CI), with adjustment for potential confounders.

**Results:**

Compared with women with non-O blood types (A, B, and AB), women with blood type O had a significantly increased risk of GDU from birth (multivariable-adjusted HR 1.18; 95% CI, 1.04–1.34). Moreover, the highest cumulative incidence of GDU was observed in women born pre-1956 with blood type O. In a subgroup analysis stratified by birth year (pre-1956 or post-1955), the multivariable-adjusted HR of women with blood type O was 1.22 (95% CI, 1.00–1.49) and 1.15 (95% CI, 0.98–1.35) in the pre-1956 and post-1955 groups, respectively.

**Conclusion:**

In this large, combined, ambispective cohort study of Japanese women, older women with blood type O had a higher risk of developing GDU than those with other blood types.

## INTRODUCTION

The ABO gene on chromosome 9q34 encodes glycosyltransferases that catalyze the transfer of nucleotide donor sugars to the H antigen to form the ABO blood group antigens.^1^ Human ABO blood group antigens are expressed on the surface of red blood cells and a variety of human cells and tissues. Several studies have suggested that the blood type may influence carcinogenesis,^2^^,^^3^ and ABO blood types have been associated with risk of several malignancies.^4^ The association between ABO blood types and risk of certain diseases of the upper gastrointestinal tract has also been investigated in several studies.^4^^,^^5^ For example, the association between ABO and peptic ulcer was one of the first to be identified,^6^ and it was shown that individuals with blood type O had a higher susceptibility to peptic and duodenal ulcers compared with individuals with other blood types.^7^

We conducted an ambispective analysis of the ABO blood group and risk of gastroduodenal ulcers (GDU) in the Japan Nurses’ Health Study (JNHS; *n* = 15,019). Our study aimed to examine the association with self-reported serologic blood type among 13,420 women, including 1,336 incident cases of GDU from birth.

## METHODS

### Study population

The JNHS is a large prospective cohort study designed to investigate the effects of lifestyle and healthcare practices on the health of Japanese women.^8^ Participants were recruited from 2001 through 2007. A total of 49,927 women from all 47 prefectures in Japan responded to the baseline survey. Among them, 15,019 women agreed to be followed up and returned signed informed-consent sheets, together with their completed baseline questionnaires. The study population included women of at least 25 years of age that practiced as registered nurses, licensed practical nurses, public health nurses, and/or midwives and resided in Japan at the time of the baseline survey. The JNHS coordination and data center is located at the Epidemiological Research Office of the School of Health Sciences at Gunma University. The JNHS study was approved by the Ethics Committee of Gunma University, Japan.

In the first follow-up questionnaire (2nd-year questionnaire), we asked participants to report their blood type (A, AB, B, O, or unknown) and Rh factor (positive, negative, or unknown). We conducted a validation study by performing serologic testing (ABO serotyping) in a subsample of 38 subjects from the Gunma Nurses’ Health Study.^9^^,^^10^ The consistency of self-reported and serologically confirmed ABO blood type was 92%. For the serologically checked ABO blood types that showed inconsistency with the self-reported ABO blood types (probably caused by the low quality of those frozen blood samples), we further confirmed the self-reported ABO blood types by performing ABO genotyping. We examined three single nucleotide polymorphisms (SNPs) (loci) on the ABO gene, namely rs8176719, rs8176746, and rs505922, using DNA sequencing. These SNPs are responsible for ABO blood group phenotypes in the Japanese populations.^11^^,^^12^ The rs8176719 and rs505922 polymorphisms are markers of the O allele, while rs8176746 is used to distinguish the B allele from the A allele. The consistency of self-reported and ABO genotyping was 100%.

**Figure 1.**
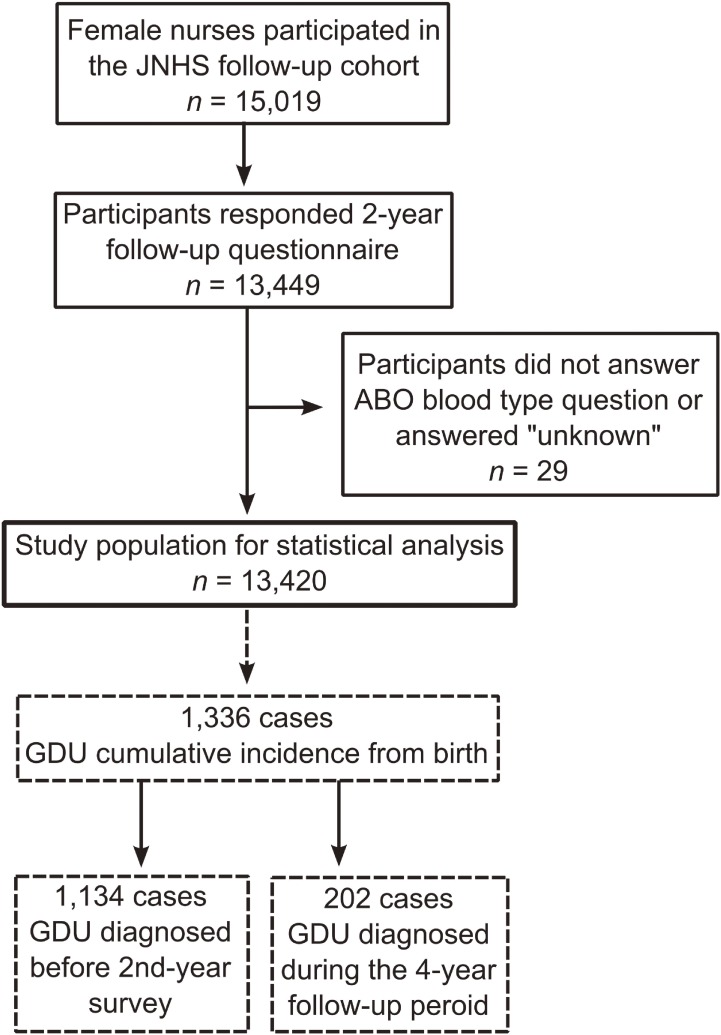
Data collection flow chart by ABO blood types information and gastroduodenal ulcer (GDU) diagnosis in the Japan Nurses’ Health Study (JNHS).

In the 2nd-year follow up questionnaire, we asked the women if they had received a diagnosis of GDU from a physician during the past 2 years. As for the 4th- and 6th-year follow-up questionnaires, we asked the women if they had ever received a diagnosis of GDU from a physician. Out of the 13,420 women, 1,336 women reported a history of physician-diagnosed GDU from birth. After excluding cases of GDU diagnosed before the 2nd-year survey, we identified 202 incident GDU cases during the 4-year follow-up period (from the 2nd-year survey to the 6th-year survey) (Figure 1). Information on lifestyle factors, such as smoking, alcohol consumption, use of aspirin/nonsteroidal anti-inflammatory drugs (NSAIDs), and dietary factors, was collected through the baseline and follow-up self-administered questionnaires.

### Statistical analysis

Differences in characteristics between ABO blood types were assessed using the chi-squared test. The effects of ABO blood types on the risk of GDU were examined using Cox regression analysis, with adjustment for potential confounders to estimate adjusted hazard ratios (HRs) and 95% confidence intervals (CIs). Potential confounders considered in the multivariable analysis were birth year (pre-1956 or post-1955), smoking status (non-smoker or current smoker), alcohol consumption (≤2 days/week or ≥3 days/week), use of NSAIDs (no or yes), and consumption of green tea (≤2 days/week or ≥3 days/week), coffee (≤2 days/week or ≥3 days/week), miso soup (≤3 days/week or ≥4 days/week), and breakfast (bread or rice) at the 2nd-year survey. Additionally, we conducted subgroup analyses stratified by birth year (pre-1956 or post-1955) because the prevalence of *Helicobacter pylori* (*H. pylori*) infection was quite different between these two age groups.^29^ Using Kaplan-Meier analysis, we plotted the cumulative incidence of GDU by blood group in women born pre-1956 and post-1955 and tested for differences using the log-rank test and the generalized Wilcoxon test.

Because GDU is not life-threatening and because ABO information is not time-dependent, the ambispective analysis combining retrospective and prospective data plays an appropriate role, as does the separate prospective analysis. We restrictively analyzed the prospective data as a sensitivity analysis, despite 4 years being a relatively short follow-up period. All statistical analyses were carried out using Statistical Analysis Software SAS version 9.4 (SAS Institute, Cary, NC, USA). All *P* values were two-sided. A *P* value <0.05 was considered statistically significant.

## RESULTS

The 13,449 participants responded to the 2-year questionnaire, including questions of ABO blood type and Rh factor type. Most (99.8%) of the respondents provided their blood type, and 98.5% of these women also provided their Rh type. We then excluded participants who did not report their blood type (*n* = 29), which resulted in an ultimate sample of 13,420 women (Figure 1).

Characteristics of the study subjects by ABO blood type in JNHS are shown in Table 1. There were no differences in the baseline characteristics of study participants by blood type. Of the women in our study population, 29.4% reported blood type O, 38.6% type A, 22.4% type B, and 9.6% type AB.

**Table 1.** Characteristics of 13,420 women by ABO blood type at the 2nd-year follow-up survey of the Japan Nurses’ Health Study, 2003–2009

	**ABO blood type**								

	O (*n*)	%	A (*n*)	%	B (*n*)	%	AB (*n*)	%	*P* value^a^
**Number of women**	3,943		5,173		3,012		1,292		
**Birth year**									
Pre-1956	1,137	28.8	1,489	28.8	903	30.0	388	30.0	0.57
Post-1955	2,806	71.2	3,684	71.2	2,109	70.0	904	70.0	
**Smoking status**									
Non-smoker	3,425	86.9	4,521	87.4	2,638	87.7	1,115	86.3	0.57
Current smoker	515	13.1	651	12.6	371	12.3	177	13.7	
Missing	3		1		3		0		
**Alcohol consumption**									
≤2 days/week	3,009	76.9	3,974	77.2	2,329	77.6	962	74.7	0.20
≥3 days/week	906	23.1	1,171	22.8	674	22.4	326	25.3	
Missing	28		28		9		4		
**Rh factor**									
Positive	3,639	93.5	4,780	93.8	2,768	93.2	1,186	92.6	0.39
Negative	253	6.5	315	6.2	202	6.8	95	7.4	
Missing	51		78		42		11		
**NSAID user**									
No	3,273	83.8	4,265	83.1	2,471	82.8	1,045	82.1	0.46
Yes	632	16.2	870	16.9	514	17.2	228	17.9	
Missing	38		38		27		19		
**Green tea consumption**									
≤2 days/week	853	21.7	1,140	22.1	639	21.3	276	21.4	0.83
≥3 days/week	3,081	78.3	4,021	77.9	2,366	78.7	1,015	78.6	
Missing	9		12		7		1		
**Coffee consumption**									
≤2 days/week	728	18.5	1,035	20.1	593	19.7	242	18.8	0.26
≥3 days/week	3,206	81.5	4,126	79.9	2,412	80.3	1,049	81.2	
Missing	9		12		7		1		
**Miso soup consumption**									
≤3 days/week	1,961	49.9	2,535	49.1	1,471	49.0	658	51.2	0.51
≥4 days/week	1,971	50.1	2,629	50.9	1,533	51.0	628	48.8	
Missing	11		9		8		6		
**Breakfast**									
Bread	1,507	41.6	1,984	41.8	1,143	41.2	450	38.0	0.13
Rice	2,116	58.4	2,767	58.2	1,631	58.8	733	62.0	
Missing	320		422		238		109		

NSAID, nonsteroidal anti-inflammatory drug; Rh, Rhesus.

^a^Chi-squared test.

The mean age at study entry was 41.9 years (median, 41; interquartile range, 13). Compared with women born post-1955, women born pre-1956 were at significantly higher risk of GDU. The cumulative incidence of GDU from birth was 13.8% (95% CI, 12.8–15.0%) and 8.4% (95% CI, 7.8–8.9%) in women born pre-1956 and post-1955, respectively.

The cumulative incidence of GDU (*n* = 1336) from birth was 10.9% (95% CI, 9.9–11.9%), 9.7% (95% CI, 8.9–10.5%), 9.1% (95% CI, 8.1–10.2%), and 10.3% (95% CI, 8.7–12.1%) in women with blood types O, A, B, and AB, respectively. The 4-year cumulative incidence of GDU (*n* = 202) was 1.5% (95% CI, 1.1–1.9%), 1.7% (95% CI, 1.3–2.1%), 1.1% (95% CI, 0.8–1.6%), and 1.9% (95% CI, 1.2–2.8%) in women with blood types O, A, B, and AB, respectively (Table 2). Additionally, we examined the cumulative incidence of GDU by birth-year group. The cumulative incidence of GDU from birth was 15.5% (95% CI, 13.4–17.7%), 13.2% (95% CI, 11.6–15.1%), 12.7% (95% CI, 10.6–15.1%), and 13.9% (95% CI, 10.6–17.8%) in women born pre-1956 with blood types O, A, B, and AB, respectively (Table 2).

**Table 2.** Cumulative incidence of ulcer from birth and during the 4-year follow-up period, Japan Nurses’ Health Study, 2001–2013

**ABO blood type**	**From birth (*n* = 1,336)**			**Four-year follow-up (*n* = 202)**		
	
	Number of Cases	Cumulative Incidence %	95% CI	Number of Cases	Cumulative Incidence %	95% CI
**O**	428	10.9	9.9–11.9	58	1.5	1.1–1.9
**Non-O**	908	9.6	9.0–10.2	144	1.5	1.3–1.8
** A**	500	9.7	8.9–10.5	86	1.7	1.3–2.1
** B**	275	9.1	8.1–10.2	34	1.1	0.8–1.6
** AB**	133	10.3	8.7–12.1	24	1.9	1.2–2.8

**By birth year**						
**Pre-1956**						

**O**	176	15.5	13.4–17.7	19	1.7	1.0–2.6
**Non-O**	366	13.2	11.9–14.5	47	1.7	1.2–2.2
** A**	197	13.2	11.6–15.1	29	2.0	1.3–2.8
** B**	115	12.7	10.6–15.1	12	1.3	0.7–2.3
** AB**	54	13.9	10.6–17.8	6	1.6	0.6–3.3

**Post-1955**						

**O**	252	9.0	8.0–10.1	39	1.4	1.0–1.9
**Non-O**	542	8.1	7.5–8.8	97	1.5	1.1–1.8
** A**	303	8.2	7.4–9.2	57	1.6	1.2–2.0
** B**	160	7.6	6.5–8.8	22	1.0	0.7–1.6
** AB**	79	8.7	7.0–10.8	18	2.0	1.2–3.1

CI, confidence interval; Non-O, A, B, and AB blood groups.

**Figure 2.**
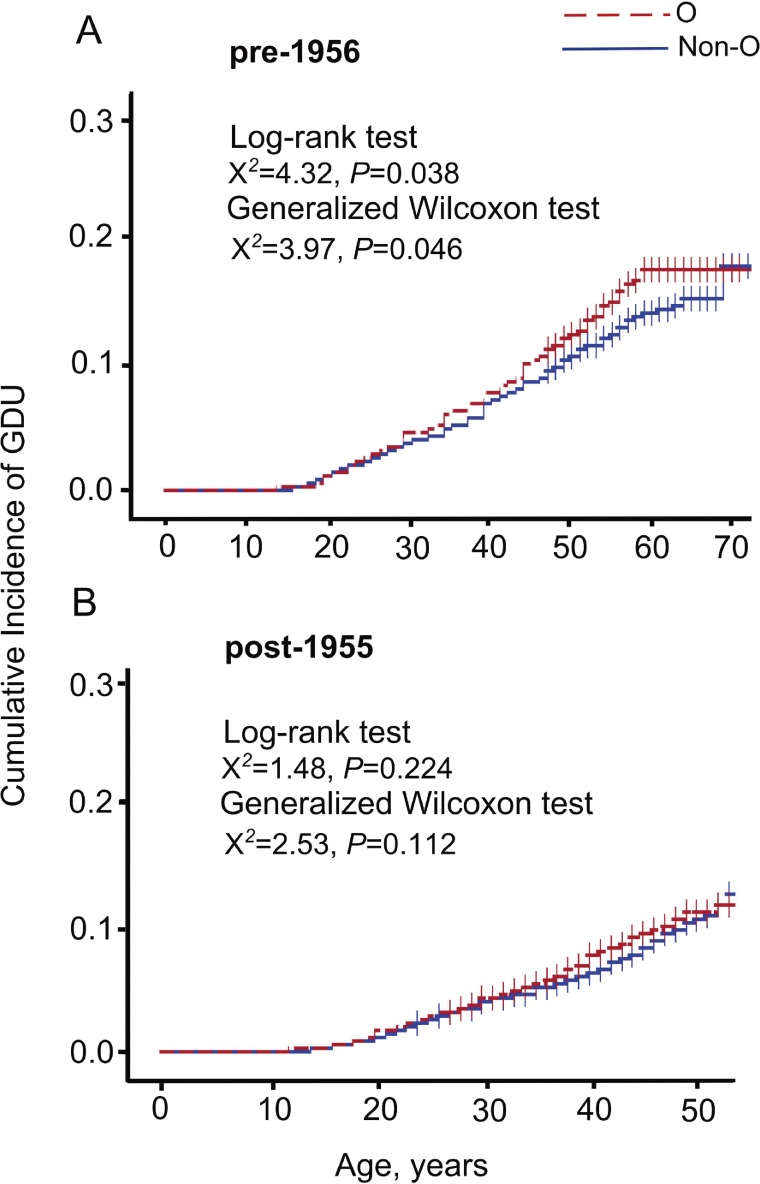
Kaplan-Meier Curves of the cumulative incidence of gastroduodenal ulcer (GDU) in women with O and non-O blood types in the Japan Nurses’ Health Study (JNHS) **A**: women born pre-1956; **B**: women born post-1955.

We examined the risk of GDU by comparing women with blood type O with women with non-O (A, B, and AB) blood types. Compared with women reporting non-O blood types, those with blood type O had a significantly higher risk of GDU (multivariable-adjusted HR 1.18; 95% CI, 1.04–1.34) (Table 3). We did not observe any significant difference of GDU risk in women with Rh-negative factor as compared with women with Rh-positive factor (multivariable-adjusted HR 0.98; 95% CI, 0.78–1.23). In the subgroup analyses stratified by birth year, the cumulative GDU incidence from birth in women with blood type O in the pre-1956 subgroup was significantly higher than it was in those with non-O blood types (log-rank test, *P* = 0.038; generalized Wilcoxon test, *P* = 0.046). However, in the post-1955 subgroup, the increased risk of GDU was not statistically significant (log-rank test, *P* = 0.224; generalized Wilcoxon test, *P* = 0.112) (Figure 2A and Figure 2B). The multivariable-adjusted HRs of women with blood type O were 1.22 (95% CI, 1.00–1.49) and 1.15 (95% CI, 0.98–1.35) in the pre-1956 subgroup and the post-1955 subgroup, respectively (Table 3).

**Table 3.** Multivariable-adjusted HR for incident gastroduodenal ulcer among 13 420 women according to ABO blood type, Rh factor, and lifestyle factors in the Japan Nurses’ Health Study (from birth)

**(a) All participants**			

	Multivariable-adjusted HR	95% CI	*P* value

**Birth year**			
Post-1955	1.00	Referent	
Pre-1956	1.04	0.91–1.19	0.59
**ABO blood group**			
Non-O	1.00	Referent	
O	1.18	1.04–1.34	<0.01

O	1.00	Referent	
A	0.88^a^	0.78–1.01	0.06
B	0.83^a^	0.72–0.97	0.02
AB	0.93^a^	0.77–1.14	0.49

**Rh factor**			
Positive	1.00	Referent	
Negative	0.98	0.78–1.23	0.85
**Smoking status**			
Non-smoker	1.00	Referent	
Current smoker	1.74	1.48–2.03	<0.01
**Alcohol consumption**			
≤2 days/week	1.00	Referent	
≥3 days/week	0.96	0.84–1.11	0.57
**NSAID users**			
No	1.00	Referent	
Yes	1.35	1.16–1.57	<0.01
**Green tea consumption**			
≤2 days/week	1.00	Referent	
≥3 days/week	1.00	0.85–1.17	0.98
**Coffee consumption**			
≤2 days/week	1.00	Referent	
≥3 days/week	0.81	0.70–0.93	<0.01
**Miso soup consumption**			
≤3 days/week	1.00	Referent	
≥4 days/week	0.93	0.83–1.06	0.28
**Breakfast**			
Rice	1.00	Referent	
Bread	0.87	0.77–0.99	0.04



**(b) Participants born pre-1956**			

	Multivariable-adjusted HR	95% CI	*P* value

**ABO blood group**			
Non-O	1.00	Referent	
O	1.22	1.00–1.49	0.047

O	1.00	Referent	
A	0.83^a^	0.68–1.02	0.07
B	0.80^a^	0.63–1.01	0.06
AB	0.87^a^	0.64–1.18	0.37

**Rh factor**			
Positive	1.00	Referent	
Negative	1.01	0.73–1.38	0.98
**Smoking status**			
Non-smoker	1.00	Referent	
Current smoker	1.89	1.45–2.48	<0.01
**Alcohol consumption**			
≤2 days/week	1.00	Referent	
≥3 days/week	0.82	0.65–1.03	0.09
**NSAID users**			
No	1.00	Referent	
Yes	1.20	0.89–1.63	0.23
**Green tea consumption**			
≤2 days/week	1.00	Referent	
≥3 days/week	0.87	0.66–1.17	0.36
**Coffee consumption**			
≤2 days/week	1.00	Referent	
≥3 days/week	0.86	0.68–1.09	0.21
**Miso soup consumption**			
≤3 days/week	1.00	Referent	
≥4 days/week	1.04	0.85–1.27	0.70
**Breakfast**			
Rice	1.00	Referent	
Bread	0.98	0.79–1.20	0.82



**(c) Participants born post-1955**			

	Multivariable-adjusted HR	95% CI	*P* value

**ABO blood group**			
Non-O	1.00	Referent	
O	1.15	0.98–1.35	0.09

O	1.00	Referent	
A	0.92^a^	0.78–1.09	0.36
B	0.86^a^	0.70–1.05	0.13
AB	0.98^a^	0.76–1.27	0.88

**Rh factor**			
Positive	1.00	Referent	
Negative	0.94	0.67–1.32	0.71
**Smoking status**			
Non-smoker	1.00	Referent	
Current smoker	1.65	1.37–2.00	<0.01
**Alcohol consumption**			
≤2 days/week	1.00	Referent	
≥3 days/week	1.06	0.89–1.27	0.50
**NSAID users**			
No	1.00	Referent	
Yes	1.41	1.19–1.68	<0.01
**Green tea consumption**			
≤2 days/week	1.00	Referent	
≥3 days/week	1.05	0.87–1.26	0.60
**Coffee consumption**			
≤2 days/week	1.00	Referent	
≥3 days/week	0.77	0.64–0.92	<0.01
**Miso soup consumption**			
≤3 days/week	1.00	Referent	
≥4 days/week	0.88	0.75–1.03	0.10
**Breakfast**			
Rice	1.00	Referent	
Bread	0.82	0.70–0.97	0.02


CI, confidence interval; Non-O, A, B, and AB blood groups; NSAID, nonsteroidal anti-inflammatory drug; HR, hazard ratio; Rh, Rhesus.

^a^Birth-year-group adjusted HR.

We then conducted a sensitivity analysis by examining the above observed association using incident GDU cases during the 4-year prospective observation. We did not find any statistically significant increased risk of GDU in women with blood type O compared with those with non-O blood types for all participants (multivariable-adjusted HR 0.93; 95% CI, 0.66–1.32), in women born pre-1956 (HR 1.06; 95% CI, 0.58–1.94), or in women born post-1955 (HR 0.88; 95% CI, 0.58–1.35).

Although it was not a primary purpose of the study, we explored whether there was any association between GDU incident cases from birth and our already confirmed cases of gastric cancer from birth (*n* = 45) using logistic regression analysis. We found that women with GDU were at significantly higher risk of developing gastric cancer (birth-year-group adjusted odds ratio 2.28; 95% CI, 1.06–4.89). We further explored the association between gastric cancer and ABO blood types, and we found a non-significant increased risk of gastric cancer among women with blood type O (odds ratio adjusted for birth-year-group and GDU 1.04; 95% CI, 0.51–2.13).

## DISCUSSION

In this prospective cohort of Japanese women, blood type A (38.6%) was more frequent than other blood types (type O, 29.4%; type B, 22.4%; and type AB, 9.6%). This frequency distribution is similar to that reported by Fujita et al,^13^ who studied the distribution of the ABO blood groups in Japan (*n* = 4,464,349 Japanese individuals). We found that the risk of GDU was significantly higher among those with blood type O than those with non-O blood types. The association between the ABO blood group and GDU was not significantly modified by other known risk factors for GDU, including smoking, alcohol consumption, NSAID use, and dietary factors. Associations between ABO blood types and the upper gastrointestinal tract diseases have been investigated in many studies. However, to the best of our knowledge, the association between ABO blood types and GDU risk had not been evaluated in a large cohort of Japanese women.

The studies by Aird et al^6^^,^^14^ were the first to report an association between the ABO blood group and both peptic ulcer and gastric cancer. They found that individuals with blood type O had a higher risk of peptic ulcers and that individuals with blood type A had a 20% increased risk of carcinoma of the stomach compared to those with other blood types. Recently, a large prospective population-based study performed within a cohort of Scandinavian blood donors included in the Scandinavian Donations and Transfusions (SCANDAT) study confirmed that blood type O is associated with a higher risk of peptic ulcers and that blood group A is associated with a higher risk of gastric cancer.^15^ Despite the evidence supporting the link between the ABO blood group and gastric cancer, a case-control analysis of data of the European Institute of Oncology did not confirm the aforementioned relationship.^16^

Regarding GDU, our results are aligned with the previous abovementioned studies. While gastric cancer did not show any association with blood type A, as reported previously, the associations tended to be similar for GDU. However, the association between blood type O and increased risk of gastric cancer did not reach statistical significance. It is possible that the lack of significance was a result of the limited numbers of gastric cancer cases (*n* = 45) in this study. Therefore, our results need to be confirmed in further studies analyzing a greater number of incident cases of gastric cancer. However, our results were supported by the significant association observed between GDU and gastric cancer. Women with GDU were at higher risk of developing gastric cancer in our study. This finding is in agreement with previous reports that found an association between benign gastric ulcers and gastric cancers and probably reflects common risk factors (ie, mainly *H. pylori* infection).^17^^,^^18^ Taken together, these results suggest that women with blood type O might have an increased susceptibility to gastroduodenal ulceration and may be at higher risk of developing gastric cancer.

The mechanisms underlying the associations between the ABO blood group and the upper gastrointestinal tract diseases remain uncertain. However, several lines of biological evidence might explain the associations observed. Alteration in the antigenic and genetic structures and expression of blood groups may influence malignant progression by changing cell motility, sensitivity to apoptosis, and immune surveillance.^2^ A genome-wide association analysis suggests that SNPs at the ABO gene locus are associated with circulating levels of tumor necrosis factor alpha, soluble intercellular adhesion molecule-1, soluble E-selectin, and soluble P-selectin.^19^^–^^22^ Thus, ABO blood group alleles may influence the systemic inflammatory state and immune response of certain malignances and diseases.^22^^–^^24^

Previously, it was speculated that individuals with blood type O have excessive gastric production of hydrochloric acid and consequently are susceptible to duodenal ulceration.^25^ Thus, the experimental results indicate that the gastric secretory cell mass is probably larger in individuals with blood type O than those with blood type A.^26^ Additionally, glycosyltransferase activity, encoded by the ABO gene, has been associated with circulating levels of von Willebrand factor (VWF) and the risk of venous thromboembolism.^23^^,^^24^ It has been reported that plasma VWF levels are 25–35% lower in subjects with blood type O than in non-O individuals.^27^ Horwich et al^28^ investigated subjects with duodenal ulcers and healthy controls and reported a significant increase of bleeding duodenal ulcers in subjects with blood type O compared with the controls.

*H. pylori* infection is considered a major risk factor of both gastric cancer and peptic ulcer.^29^ Because of the high prevalence of *H. pylori* infection in the Japanese population, the incidence of peptic ulcer and gastric cancer is much higher in Japanese individuals than in individuals of European descent.^30^ *H. pylori* infection rates vary with age, with higher rates among individuals born before 1950 and lower for those born thereafter, owing to the massive improvement in hygiene and the economic environment in Japan in the post-war decades.^29^ Our results showed that women born before 1956 were at significantly higher risk of GDU than were those born after 1955, which is consistent with the previously established association between *H. pylori* infection, birth year, and the incidence of GDU in the Japanese population. Furthermore, we found that women with blood type O born pre-1956 had a higher cumulative incidence of GDU than those with blood type O born post-1955. These findings seem to explain why the older generation of Japanese women, especially those with blood type O, had a higher incidence of GDU.

The mechanism of the association between *H. pylori* infection and blood groups has been reported in several studies.^31^^–^^34^ It was reported that the increased susceptibility of subjects with blood type O to develop peptic ulcer might be attributable to the higher density of *H. pylori* colonization and higher inflammatory responses to *H. pylori* compared with individuals with other blood types.^35^ It has also been shown that epithelial cells of individuals with blood type O bound significantly more to *H. pylori* than epithelial cells of individuals with other blood types.^32^ Interestingly, it has been reported that the blood group antigen-binding adhesion has been shown to mediate adherence of *H. pylori* to Lewis b receptors in the gastric epithelium.^33^^,^^34^ These fucosylated blood group antigens are highly expressed in the gastrointestinal epithelium, as well as on the surface of red blood cells, endothelium, kidney, and genitourinary epithelium.^36^^,^^37^ Collectively, these findings suggest that the increased risk of women with blood type O to develop GDU observed in our study might be attributable to the higher *H. pylori* colonization and to the mediated adhesion of *H. pylori* to the gastroduodenal epithelium observed in individuals with blood type O.

The strengths of the present study include a large study population, a high proportion of followed-up participants, and the availability of detailed lifestyle data, which allowed us to examine confounding and effect modification by several exposures of interest. Although the use of self-reported blood type may have introduced some exposure misclassification, the high concordance between self-reported blood type and serologic/genotypic testing in a subset of participants suggests that these health professionals report their blood type with a high degree of accuracy. The limitations of the present study relate to the effect of *H. pylori* infection. This issue was not investigated in our study because we lacked information on such infection for all subjects. Our cohort questionnaire did not ask the women to specify whether they had gastric or duodenal ulcers. Moreover, no significantly increased risk of GDU was evident in women with blood type O in the prospective analysis. This finding might have resulted from the small number of incident GDU cases in the relatively short 4-year follow-up period and/or the possible effect of more common administration of bacterial elimination therapy for *H. pylori* infection in the past decade.

In summary, the results from this ambispective analysis of a cohort of Japanese women (the JNHS cohort) showed that women, particularly those born before 1956, with blood type O (independent of other important risk factors in the Japanese population) have an increased susceptibility to gastroduodenal ulceration.
